# Prevalence and Correlates of Perinatal Depression and Anxiety at Perinatal Clinics in Southwestern Uganda: A Cross-Sectional Study

**DOI:** 10.7759/cureus.72241

**Published:** 2024-10-23

**Authors:** Gladys Nakidde, Edward Kumakech, John F Mugisha

**Affiliations:** 1 Obstetrics and Gynaecology, Pan African University Life and Earth Sciences Institute, University of Ibadan, Ibadan, NGA; 2 Nursing, Bishop Stuart University, Mbarara, UGA; 3 Nursing and Midwifery, Lira University, Lira, UGA; 4 Public Health, Pan African University Life and Earth Sciences Institute, University of Ibadan, Ibadan, NGA; 5 Public Health, Bishop Stuart University, Mbarara, UGA

**Keywords:** maternal mental health, perinatal anxiety, perinatal depression, predictors, uganda

## Abstract

Introduction

Depression and anxiety during pregnancy and after childbirth are a considerable global public health burden, but data on their magnitude - which is critical for effective intervention - are limited in Uganda. We investigated the prevalence and risk factors for perinatal depression and anxiety among women who visited perinatal clinics in southwestern Uganda.

Methods

From June to August 2022, a cross-sectional study was conducted on women attending perinatal clinics at 20 different healthcare facilities in southwestern Uganda. Participants were chosen by multi-stage clustered sampling. To screen for depression, the Edinburgh Postnatal Depression Scale was used, while anxiety symptoms were evaluated using the Generalized Anxiety Disorder-7 Scale. Multivariate logistic regression was performed to identify the risk factors for perinatal depression and anxiety.

Results

A total of 517 participants were enrolled. The overall prevalence of perinatal depression was 10.6%, while anxiety was 1.7%. The prevalence of antepartum depression (APD) and postpartum depression (PPD) was 9.9% and 10.8%, respectively, while the prevalence of antepartum anxiety (APA) and postpartum anxiety (PPA) was 1.0% and 3%. Adequate partner support was protective against APD (p = 0.036), and a history of stressful situations predicted APD symptoms (p = 0.001). A history of mental illness increased the likelihood of PPD symptoms (p = 0.004). There were no statistically significant risk factors for perinatal anxiety.

Conclusion

Perinatal depression and anxiety were highly prevalent and primarily associated with psychosocial factors in Uganda. Thus, psychosocial screening and the incorporation of social strategies into routine perinatal care services may be highly beneficial.

## Introduction

Perinatal depression and anxiety are a major global health concern that have received little attention, especially in low- and middle-income countries (LMICs) [1]. The worldwide mean prevalence of perinatal depression is about 10% during pregnancy and 13% in the postpartum period, but these figures are higher - up to 16.6% during pregnancy and 19.8% in the postpartum period - in LMICs such as Uganda [2,3]. On the other hand, the prevalence of perinatal anxiety ranges between 1% and 26% in both low- and high-income economies [4]. In Uganda, a handful of studies conducted mainly in the central and northern regions of the country reported the prevalence of postpartum depression between 6.1% and 43% [5,6], antenatal depression between 35.8% and 37.7% [7,8], and perinatal anxiety at 13% [9].

Perinatal depression and anxiety are associated with many prenatal and postnatal adverse effects, including the impaired ability of the mother to care for herself and to seek perinatal medical care; abuse of drugs like alcohol; poor appetite leading to premature deliveries and low birth weight; poor mother-child bonding; maternal mood swings and hostility towards the child; infanticide; and maternal suicide, among others [5,10]. Interestingly, the diagnostic coverage of perinatal depression and anxiety is reportedly less than 10% in LMICs, even though these are manageable conditions if detected and appropriate treatment is initiated early [8,11]. The low level of diagnostic coverage could be attributed to a lack of knowledge/research and skills among health workers and community members about the prevalence, risk factors, diagnosis, and treatment of these conditions, which is critical for developing effective care interventions. For example, in Uganda, there are no specific clinical guidelines for screening maternal mental health as a standard practice in maternity centers, according to the Uganda Clinical Guidelines, 2023. Given the importance of early diagnosis and treatment of perinatal mental illness, this study aimed to determine the prevalence and correlation of perinatal depression and anxiety in southwestern Uganda. The findings of this study may be used to guide perinatal depression and anxiety care practices and policy guidelines in Uganda and other similar settings.

## Materials and methods

Study design and setting

This was a cross-sectional quantitative study conducted at 20 public healthcare facilities in the Mbarara and Kabale districts, southwestern Uganda. The two districts were purposively selected because they have the largest combined catchment population, ensuring adequate regional representation. Kabale and Mbarara districts are approximately 420 and 250 km, respectively, southwest of Kampala, Uganda's capital city. Uganda's health care system is organized into seven levels: health center I (HC I) to HC IV, general hospitals, regional referral hospitals, and national referral hospitals, depending on personnel training, services, and target population. The hospital offers the highest level of care, including specialist services, research, and consultations. Complicated cases are referred to the next level until the highest level is reached. General hospitals serve 500,000; regional hospitals serve 2,000,000; and national hospitals serve 10,000,000. Maternity care services begin at HC III; hence, this study focused on care facilities from HC III [12].

Study population and sample size determination

The study population was made up of women of reproductive age who sought perinatal care at participating healthcare facilities during the data collection period and volunteered to take part in the study. All women with pre-existing mental health conditions and/or taking antipsychotic medication were excluded to minimize inaccuracies that would have resulted from capturing mental illness prevalence outside the perinatal period. The minimum sample size was calculated using the Kish and Leslie formula for proportions [13]: n = Z²pq/d², where n = sample size; Z = Z score for the desired confidence level (1.96 for a 95% confidence interval); d = the marginal error = 0.05; p = the estimated proportion of the prevalence of perinatal mental illness present in the population = 43% from a cross-sectional study on postpartum mothers assessing postpartum depression using the Edinburgh Postnatal Depression (EPD) Scale = 0.43 [5]; and q = 1 - p = 1 - 0.43 = 0.57. Therefore, n = (1.96² * 0.43 * 0.57)/(0.05²) = 377. Considering the cluster effect due to the nature of the study, with various populations as clusters from different health facility levels, we applied the design effect of 1.5, resulting in a final sample size of n = 566.

Sampling and data collection procedure

Systemic cluster sampling was utilized to select study sites and participants as follows: only two regional referral hospitals and six HC IVs in the study districts were selected. Then, among a total of 24 HC IIIs in the participating districts, 12 were selected at random. The clusters were organized into three levels: district (Mbarara and Kabale), health facility (Referral, HC IV, and HC III), and maternity department (antenatal and postnatal). Participants were recruited at the participating healthcare facilities using a consecutive sampling method, ensuring that all eligible and consenting women were included in the study. All women who consented to participate were interviewed for 30-45 minutes using a researcher-administered semi-structured questionnaire.

Data collection tools

Data were collected using a researcher-administered semi-structured questionnaire. The questionnaire had three sections: (i) sociodemographic characteristics, (ii) clinical and obstetric characteristics, and (iii) screening tools for maternal depression and anxiety - the EPD Scale and the Generalized Anxiety Disorder 7-Item (GAD-7) Scale, respectively. The EPD Scale has 10 items assessing the occurrence of events in the previous week. Each item has the lowest score of ‘0,’ meaning absent symptoms for depression, and the highest score of ‘3,’ meaning the individual has the severe form of the symptom. The maximum score is 30, and any score of 10 and above qualifies a woman as having depression symptoms [14].

The GAD 7-Item Scale was used to screen for anxiety. Its internal consistency was good (Cronbach’s alpha = 0.722) with an intra-class coefficient of 0.70 (95% CI = 0.659-0.738).

The GAD 7-Item Scale has seven items and requires the individual woman to rate her anxiety symptoms over the previous two weeks. The items are rated on a Likert Scale ranging from 0 (not at all), 1 (several days), 2 (more than half the days), and 3 (nearly every day). A mother who scores 10 and above is considered to have moderate to severe anxiety symptoms.

Research ethics

This is a sub-study for the PhD study that was approved by the Institute for Advanced Medical Research and Training, College of Medicine, University of Ibadan Ethical Review Board (reference: UI-EC-22-008), Mbarara University of Science and Technology Research Ethics Committee (MUST-2022-414), the supervising Research Ethics Committee, and the Uganda National Council of Science and Technology (SS1362ES) in Uganda, where data collection took place. All participants provided informed consent before participation.

Data management and analysis

Data from the questionnaires was entered, coded, cleaned, and analyzed with IBM SPSS Statistics for Windows, Version 26 (Released 2019; IBM Corp., Armonk, NY, USA). The prevalence of perinatal depression and anxiety was estimated by comparing participants' scores to conventional assessment scales, specifically the EPD Scale and GAD 7-Item Scale. Binary variables were constructed using the EPD Scale tool cut-off of ≥10 for depression and the GAD 7-Item Scale cut-off of 10 for anxiety. The results, including participant characteristics, were reported as proportions and frequencies in tables and figures. Factors associated with perinatal depression and anxiety were identified, and given the categorical outcomes of the associated factors, a Chi-square or Fisher's exact test was obtained in bivariate analysis at a statistical significance level of p-value = 0.05 and a 95% CI. The multivariate analysis model incorporated all factors that were statistically significant during the bivariate analysis. In multivariate analysis, binary logistic regression was conducted after adjusting for confounders. p-values, odds ratios (ORs), and 95% CI were reported.

Quality control

The data were collected using validated tools. The EPD Scale was validated for use in maternity care settings in Uganda [14], while the GAD 7-Item Scale was validated for use in many similar settings in Africa [15] and had previously been used to collect data in Uganda [16]. The study questionnaire was pretested, and data were gathered by well-trained and experienced research assistants who spoke both English and the local languages fluently. 

## Results

A total of 517 eligible women participated, resulting in a 91% response rate. The majority of participants were aged 35 years and below (89%), and only 42.7% of participants and 33.8% of their partners had completed at least a primary school education, as shown in Table 1.

**Table 1:** Sociodemographic and obstetric characteristics of perinatal women ANC: Antenatal clinic; PNC: Postnatal clinic; YCC: Young child clinic

Participants’ characteristics, n = 517	Frequency (%)
Health facility level	
Health center III	163 (31.5)
Health center IV	96 (18.6)
Referral hospital	258 (49.9)
Age categories in years	
15-24.9	242 (46.8)
25-34.9	218 (42.2)
35-44.9	55 (10.6)
45 and above	2 (0.4)
Religion	
Catholic	189 (36.6)
Protestant	254 (49.1)
Muslim	27 (5.2)
Seventh-day Adventist	3 (6)
Pentecostal	43 (8.3)
Jehovah’s Witness	1 (0.2)
Education level	
No formal education	84 (16.2)
Primary school	221 (42.7)
Secondary school	143 (27.7)
Tertiary education	69 (13.3)
Employment status	
Unemployed/housewife	282 (54.5)
Family business self	187 (36.2)
Civil servant	48 (9.3)
Marital status	
Single	21 (4.1)
Married	486 (94.0)
Divorced or separated	10 (1.9)
Male partner's education level	
No formal education	79 (15.3)
Primary school	175 (33.8)
Secondary school	154 (29.8)
Post-secondary education	109 (21.1)
Partner's support	
Little support	47 (9.1)
Adequate support	470 (90.9)
Residence	
Rural	245 (47.4)
Urban	272 (52.6)
Number of children	
None	116 (22.4)
4-Jan	365 (70.6)
5 and above	36 (7.0)
History of intimate partner violence	
No	357 (68.7)
Yes	162 (31.3)
History of stressful situations	
No	240 (46.4)
Yes	277 (53.6)
History of mental illness	
No	453(87.6)
Yes	64 (12.4)
Perinatal department	
Antenatal department	314 (60.7)
Postnatal (PNC and YCC)	203 (39.3)
Woman's perinatal condition	
Pregnant	314 (60.7)
Delivered	203 (39.3)
Duration post-delivery, n = 203	
Up to 6 weeks	190 (93.6)
More than 6 weeks	13 (6.4)
Gestation age if pregnant, n = 314 (60.7%)	
First trimester	63 (12.2)
Second trimester	103 (19.9)
Third Trimester	148 (28.6)
Experienced complications during pregnancy, n = 314	
No	223 (71.0)
Yes	91 (29.0)
Comorbidities during pregnancy, n = 314	
No	241 (76.8)
Yes	73 (23.2)
Experienced complications postpartum, n = 203	
No	162 (79.8)
Yes	41 (20.2)
Mode of delivery, n = 203	
Vaginal delivery	111 (54.7)
Cesarean delivery	92 (45.3)
Health status after delivery, n = 203	
Unstable	12 (5.9)
Fair	68 (33.5)
Good health state	123 (60.6)
Whether the infant breastfeeds well, n = 202	
Not at all	8 (4.0)
Yes sometimes	10 (5.0)
Yes all the time	184 (91.0)
Birth weight in Kg	
<2.5	11 (5.4)
2.5-3.0	57 (28.1)
>3.0	123 (60.6)
Not weighed at birth	12 (5.9)
Whether the infant has any health problems, n = 202	
No	170 (84.2)
Yes	32 (15.8)

Prevalence of perinatal depression and anxiety

As seen in Table 2, perinatal depression prevalence was 10.6% on the EPD Scale, and perinatal anxiety was 1.7% on the GAD 7-Item Scale. The prevalence of antenatal depression was 9.9% (95% CI = 0.099 (0.068-0.137)), while postnatal depression was 10.8% (95% CI = 0.108 (0.069-0.159)). Antenatal anxiety was 1.0%, while postnatal anxiety was 3%.

**Table 2:** Prevalence of perinatal depression and anxiety among participants ANC: Antenatal clinic; PNC: Postnatal clinic; f: Frequency

Depression			Anxiety		
S/N	Variables	f (%)	S/N	Variables	f (%)
1	I have been able to laugh and see the funny side of things, N = 517		1	Feeling nervous, anxious, or on edge	
	As much as I always could	357 (69.1)		Not at all	310 (60.0)
	Not quite so much now	129 (25.0)		Several days	185 (35.8)
	Definitely not so much now	25 (4.8)		More than half the days	19 (3.7)
	Not at all	6 (1.2)		Nearly every day	3 (0.6)
2	I have looked forward with enjoyment to things		2	Not being able to stop or control worrying	
	As much as I ever did	366 (70.8)		Not at all	292 (56.5)
	Rather less than I used to	117 (22.6)		Several days	194 (37.5)
	Definitely less than I used to	27 (5.2)		More than half the day	20 (3.9)
	Hardly at all	7 (1.4)		Nearly every day	11 (2.1)
3	I have blamed myself unnecessarily when things went wrong		3	Worrying too much about different things	
	No, never	97 (18.8)		Not at all	311 (60.2)
	Not very often	306 (59.2)		Several days	169 (32.7)
	Yes, some of the time	109 (21.1)		More than half the days	31 (6.0)
	Yes, most of the time	5 (1.0)		Nearly every day	6 (1.2)
4	I have been anxious or worried for no good reason		4	Trouble relaxing	
	No, not at all	295 (57.1)		Not at all	404 (78.1)
	Hardly ever	127 (24.6)		Several days	95 (18.4)
	Yes, sometimes	84 (16.2)		More than half the days	12 (2.3)
	Yes, very often	11 (2.1)		Nearly every day	6 (1.2)
5	I have felt scared or panicky for no very good reason		5	Being so restless that it is hard to sit still	
	No, not at all	312 (60.3)		Not at all	400 (77.4)
	No, not much	146 (28.2)		Several days	95 (18.4)
	Yes, sometimes	54 (10.4)		More than half the days	18 (3.5)
	Yes, quite a lot	5 (1.0)		Nearly every day	4 (0.8)
6	Things have been getting on top of me		6	Becoming easily annoyed or irritable	
	No, I have been coping as well as ever	314 (60.7)		Not at all	246 (47.6)
	No, most of the time I have coped quite well	166 (32.1)		Several days	171 (33.1)
	Yes, sometimes I haven’t been coping as well as usual	35 (6.8)		More than half the days	74 (14.3)
	Yes, most times I haven’t been able to cope at all	2 (0.4)		Nearly every day	26 (5.0)
7	I have been so unhappy that I have had difficulty sleeping		7	Feeling afraid as if something awful might happen	
	No, not at all	339 (65.6)		Not at all	322 (62.3)
	Not very often	122 (23.3)		Several days	147 (28.4)
	Yes, sometimes	50 (9.7)		More than half the days	40 (7.7)
	Yes, most of the time	6 (1.2)		Nearly every day	8 (1.5)
8	I have felt sad or miserable		-		
	No, not at all	352 (68.1)			
	Not very often	128 (24.8)			
	Yes, quite often	30 (5.8)			
	Yes, most of the time	7 (1.4)			
9	I have been so unhappy that I have been crying				
	No, never	375 (72.7)			
	Only occasionally	109 (21.1)			
	Yes, quite often	25 (4.8)			
	Yes, most of the time	7 (1.4)			
10	The thought of harming myself has occurred to me				
	Never	475 (91.9)			
	Hardly ever	18 (3.5)			
	Sometimes	24 (4.6)			
Classification of mental illness		F (%)	OR (95% CI)		
Depression assessment			0.106 (0.081-0.136)		
Negative		462 (89.4)	-		
Positive		55 (10.6)			
Anxiety assessment			-		
1-10 (negative)		508 (98.3)	-		
≥11 (positive for anxiety)		9 (1.7)			
Depression in ANC, n = 314			0.099 (0.068-0137)		
No depression in ANC women		283 (90.1)	-		
Depression positive in ANC women		31 (9.9)			
Depression in PNC, n = 203			0.108 (0.069-0.159)		
No depression in PNC women		181 (89.2)	-		
Depression positive in PNC women		22 (10.8)			
Anxiety in ANC			0.010 (0.002-0.028)		
No anxiety in ANC		311 (99.0)	-		
Anxiety positive in ANC		3 (1.0)			
Anxiety in PNC			0.030 (0.011-0.063)		
No anxiety		197 (97.0)	-		
Anxiety positive in PNC		6 (3.0)			

Predictors of perinatal depression and anxiety

As seen in Table 3, statistically significant factors from the bivariate analysis (p-value less than 0.005) were considered for multivariate analysis, and binary logistic regression was used to obtain the predictors of antepartum depression (APD). At the multivariate level, having adequate partner support was protective against APD (p = 0.036, aOR: 3.715 (95% CI: 1.087-12.701)), and participants with a history of stressful situations were more likely to screen positive for APD (p = 0.001, aOR: 0.076 (95% CI: 0.016-0.346)).

**Table 3:** Bivariate and multivariate analysis for antepartum depression The asterisks (*) indicate statistically significant ANC: Antenatal care; IPV: Intimate partner violence; aOR: Adjusted odds ratio

Bivariate analysis of depression in ANC					Multivariate analysis of depression in ANC	
Variable	No depression (90.1%)	Depression (9.9%)	OR (95% CI)	p-value	aOR (95% CI)	p-value
Marital status				<0.001*	-	
Single	8 (2.5)	5 (1.9)	-		1	-
Married	273 (86.9)	23 (7.3)			1.332 (0.116-15.350)	0.818
Divorced	2 (0.6)	2 (0.6)			0.455 (0.047-4.414)	0.497
Partner's support			0.153 (0.065-0.360)	<0.001*	-	
Little support	22 (7.0)	11 (3.5)	-		1	-
Adequate support	261 (83.1)	20 (6.4)			3.715 (1.087-12.701)	0.036*
History of IPV			2.926 (1.379-6.208)	0.004*	-	
No	200 (63.7)	14 (4.5)	-		1	-
Yes	83 (26.4)	17 (5.4)			0.795 (0.332-1.902)	0.606
Stressful situation			6.824 (3.939-71.855)	<0.001*	-	
No	152 (48.4)	2 (0.6)	-		-	-
Yes	131 (41.7)	29 (9.2)			0.076 (0.016-0.346)	0.001*

Partner support, history of intimate partner violence, history of stressful situations, and history of mental illness had a relationship with postpartum depression (PPD). However, at the multivariate level of analysis, only participants with a history of mental illness were more likely to screen positive for PPD (p = 0.004; aOR: 0.173 (95% CI: 0.053-0.561)), as shown in Table 4.

**Table 4:** Bivariate and multivariate analysis of postpartum depression The asterisks (*) indicate statistically significant aOR: Adjusted odds ratio; IPV: Intimate partner violence; MI: Mental illness

Bivariate analysis for postpartum depression					Multivariate analysis	
Variable	No (%)	Yes (%)	OR (95% CI)	p-value	aOR (95% CI)	p-value
Marital status				0.006*	-	
Single	4 (2.0)	3 (1.5)	-		1	-
Married	173 (85.2)	17 (8.4)			5.494 (0.319-94.548)	0.241
Divorced	4 (2.0)	2 (1.0)			0.831 (0.054-12.810)	0.894
Partners’ support			0.178 (0.054-0.592)	0.010*	-	
Little support	9 (4.4)	5 (2.5)	-		1	-
Adequate support	172 (84.7)	17 (8.4)			1.342 (0.149-12.049)	0.793
History of IPV			3.144 (1.278-7.735)	0.014*	-	
No	131 (64.5)	10 (4.9)	-		1	-
Yes	50 (24.6)	13 (5.9)			0.451 (0.152-1.341)	0.152
Stressful situation			5.364 (1.533-18.764)	0.005*	-	
No	83 (40.9)	3 (1.5)	-		1	-
Yes	98 (48.3)	19 (9.4)			0.318 (0.078-1.301)	0.111
History of MI			5.893 (2.149-16.162)	0.001*	-	
No	165 (81.3)	14 (6.9)	-		1	-
Yes	16 (7.9)	8 (3.9)			0.173 (0.053-0.561)	0.004*

From Table 5, marital status, history of stressful situations, and mode of delivery were statistically significant in bivariate analysis, but not in multivariate analysis.

**Table 5:** Bivariate for perinatal anxiety among women The asterisks (*) indicate statistically significant

Variable		Bivariate analysis for factors for antepartum anxiety						
		No (99.0%)		Yes (1%)	OR (95% CI)		p-value	
Education level							0.441	
None		52 (16.6)		0	-			
Primary school		131 (41.7)		3 (1.0)				
Secondary school		87 (27.7)		0				
Post-secondary		41 (13.1)		0				
Employment status							0.487	
Unemployed		168 (53.5)		3 (1.0)	-			
Family business		112 (35.7)		0				
Civil servant		31 (9.9)		0				
Marital status							0.151	
Single		13 (4.1)		1 (0.3)	-			
Married		294 (93.6)		2 (0.6)				
Divorced		4 (1.3)		0				
Partner's support					0.229 (0.020-2.601)		0.284	
Little support		32 (10.2)		1 (0.3)	-			
Adequate support		279 (88.9)		2 (0.6)				
Stressful situation					1.937 (0.174-21.579)		1	
No		153 (48.7)		1 (0.3)	-			
Yes		158 (50.3)		2 (0.6)				
Pregnancy complications					4.944 (0.443-55.212)		0.205	
No		220 (70.5)		1 (0.3)	-			
Yes		89 (28.5)		2 (0.6)				
Postpartum anxiety								
Marital status								0.019*
Single	5 (2.5)		2 (1.0)			-		
Married	186 (91.6)		4 (2.0)					
Divorced	6 (3.0)		0					
Type of marriage, n = 190						20.250 (2.023-202.668)		0.009*
Monogamous	162 (85.3)		1 (0.5)			-		
Polygamous	24 (12.6)		3 (1.6)					
Stressful situation						1.054 (1.011-1.099)		0.040*
No	86 (42.4)		0			-		
Yes	111 (54.7)		6 (3.0)					
Mode of delivery						1.058 (1.007-1.112)		0.018*
Vaginal delivery	110 (54.7)		0			-		
Cesarean delivery	86 (42.8)		5 (2.5)					

## Discussion

This sub-study aimed to determine the prevalence and correlation of perinatal depression and anxiety among women attending perinatal clinics in southwestern Uganda. The results showed that the general prevalence of perinatal depression was 10.6%, and anxiety was 1.7%. The prevalence of APD and PPD was 9.9% and 10.8%, respectively, whereas anxiety was 1.0% and 3%. Male partner support, a history of stressful conditions, and mental illness were all significant predictors of perinatal depression. There were no statistically significant predictors of prenatal anxiety in multivariate analysis.

Our study revealed a 10.6% mean prevalence of perinatal depression, which was significantly lower than the 20.7% (95% CI, 18.4-23.0%) pooled prevalence of 50 studies from seven LMICs [17]. However, the prevalence of perinatal depression in this study was fairly close to the estimated global range of 12% to 15% [18,19]. In contrast to previous studies in Uganda, the prevalence of PPD in this study (10.8%) was greater than 6.1% [20], but significantly lower than 27.1% [21], 43% [5], and 15.9% [6]. In comparison to other African studies, it was closer to 12% in perinatal women experiencing intimate partner violence in Tanzania [22], but far lower than 50.3% in South Africa [23] and 63.7% in Rwanda [24]. In terms of APD, the prevalence in this study was 9.9%, which was well below the estimated global range of 15% to 65% [2]. However, when compared to similar studies conducted in Uganda and the region, the prevalence of APD was close to 11.8% in Ethiopia [25], but significantly lower than 37.68% [7] and 35.8% [8] in Uganda. The wide variation in perinatal depression and anxiety prevalence values observed between this study and other studies in Uganda and outside Uganda could be attributed to a variety of factors, including different assessment methods, study populations, and settings, sample sizes, timing of the perinatal period, and the research knowledge, skills, and experience of research teams [19]. For example, with the exception of the Atuhaire et al. [21] and Nakku et al. [20] studies in Uganda, which used standard diagnostic tools, that is, the Diagnostic Statistical Manual for Mental Health Problems-V (DSMV) and the Mini International Neuropsychiatric Interview (M.I.N.I.), the rest of the Ugandan studies, including our study, used screening tools that are less sensitive and specific. Furthermore, the prevalence results can vary with the EPD Scale cut-off levels, with those using EPD Scale ≥10 having a lower prevalence than those using higher cut-offs, such as EPD Scale ≥13 [18]. With regard to the study population, studies that focused on potentially vulnerable perinatal women, such as women with a history of intimate partner abuse [22], HIV infection [8], and history of baby loss [26], would most likely have a higher prevalence for perinatal depression and anxiety. Another factor that may influence the prevalence value is the sample size. The bigger the sample size, the more power the study has, and hence the more accurate the estimated prevalence value. Furthermore, the study setting and the experience, skills, and knowledge of research assistants can influence the study results. The prevalence of perinatal depression may vary depending on the perinatal period during which the study is conducted. For example, perinatal depression is reportedly lower in the first month following childbirth [19], and highest in the last trimester [27]. When compared to study findings from high-income nations, our study and studies from LMICs, in general, show a higher prevalence of perinatal depression [2,3]. This may be due to many reasons, such as cultural differences. Many cultures in LMICs forbid women from expressing their emotions or sentiments in public, which may contribute to an increased incidence of prenatal depression [18].

With regard to perinatal anxiety, the overall mean prevalence in our study was 1.7%, with antepartum and postpartum rates of 1% and 3%, respectively. These prevalence results are within the global perinatal anxiety range of 1% to 26% [4]. However, our findings were significantly lower than the prevalence rates reported in prior research, such as 13% perinatal anxiety in central Uganda [9], 24.8% antenatal anxiety in Greece [28], and 28.2% and 48.1% antenatal and postnatal anxiety in Rwanda [24]. Variability in prevalence rates could be related to different evaluation methodologies, study populations and contexts, as well as sample sizes. For example, in our study, we employed the GAD 7-Item Scale in southwestern Uganda, whereas Govender et al. used the Hamilton Anxiety Rating Scale for Antenatal Anxiety (HAMA-A) in central Uganda [9]. In terms of population and study setting, vulnerable populations, such as prime gravidas, may experience greater anxiety. Hospital-based studies may have a different prevalence from community-based studies. Furthermore, certain populations are more vulnerable than others. For example, the Micha et al. study focused on perinatal anxiety during the COVID-19 pandemic, a time when anxiety was high, which could have influenced the prevalence results [28].

With regard to the correlation between perinatal depression and anxiety, the current study did not find statistically significant risk factors for perinatal anxiety in multivariate analysis. However, numerous previous studies identified low-income levels, problematic spousal relationships, and a history of hypertension in previous pregnancies as strongly linked to antenatal anxiety [28,29]. These studies were conducted in different settings and used different assessment tools, which could have led to variable outcomes. Furthermore, this study identified stressful situations, poor spousal support, and a history of mental illness as significant risk factors for perinatal depression. These results align with findings from Ngocho et al.’s study [30]. These findings emphasize the necessity for maternity care providers to pay extra attention to perinatal women with a history of inadequate partner support, stressful situations, or a history of past perinatal mental illness due to their increased risk of developing these illnesses. The findings also highlight the importance of psychosocial factors in maternal mental health, implying the need to incorporate psychosocial screening and interventions into the training and practice of maternity care providers to ensure that at-risk mothers are identified and treated appropriately in order to reduce disease burden. Overall, the findings underline the importance of a multidisciplinary strategy that includes mental health care, social services, and partner participation.

Strengths and limitations

This study had sufficient power to determine the prevalence and risk factors for prenatal depression and anxiety. However, we assessed depression and anxiety using self-report screening instruments (EPD Scale and GAD 7-Item Scale) rather than diagnostic tools (DSM-V), which may have influenced the reported prevalence. Nevertheless, the EPD Scale is a validated tool in Uganda with known high sensitivity and specificity. The GAD 7-Item Scale was validated in West Africa, and we obtained its internal consistency with a good Cronbach alpha of 0.722. Second, because this was a healthcare facility-based cross-sectional study, we excluded participants who did not seek healthcare services at the time of data collection. This may have influenced the reported prevalence. Finally, this study used a cross-sectional methodology, which makes it impossible to evaluate the incidence points or trajectory of perinatal depression and anxiety because cross-sectional studies only collect point prevalence data. However, we excluded perinatal women with pre-existing mental illnesses prior to getting pregnant, which increases the accuracy of the study results.

Future research recommendations

There is a need for future longitudinal and qualitative investigations to gain better insights into the nature of the correlates and progression of perinatal depression and anxiety, as well as the development of more effective intervention policies and guidelines.

## Conclusions

Perinatal depression prevalence is higher than that of perinatal anxiety. The prevalence of depression and anxiety is slightly higher in postpartum periods than in antepartum periods. Male partner support, a history of stressful situations, and mental illness were significant predictors of perinatal depression. There were no statistically significant predictors of perinatal anxiety in multivariate analysis; however, at the bivariate level, marital status, type of marriage, stressful situations, and mode of delivery were statistically significant. Since perinatal depression and anxiety are significantly related to interpersonal and social factors, this highlights the need to incorporate social interventions into strategies to improve maternal mental health, as well as to prioritize routine psychosocial screening in perinatal clinics, especially for women with a history of poor partner support, stressful situations, and mental illness. 

## Appendices

Appendix I (questionnaire for antepartum and postpartum women​​​​​​​)

Questionnaire Number: ……/……

Date: ………….…

ID Number: ………….…

1) Sociodemographic Chararcteristics

**Table 6:** Questionnaire for prevalence and correlation for maternal mental health problems in southwestern Uganda

S/N	Parameter	Response
1.	Department	Antenatal Care, Postnatal Ward, Young Child Clinic (YCC)
2.	Age in years	……………………………………………………..
3.	Religion	Catholic, Protestant, Muslim, Seventh-Day Adventist, Other (specify)
4.	Level of education	No formal education, Completed primary school, Completed secondary school, Completed post-secondary education
5.	Employment	Employed in formal sector (civil servant), Employed in small family business (self-employed), Unemployed or housewife
6.	Marital status	Married, Single, Divorced or Separated
7.	If married, type of marriage	Monogamous marriage, Polygamous marriage
8.	Number of other female partners in sexual relationship with male partner	Only me, One or two other women, Currently single/divorced/separated
9.	Male partner’s level of education	No formal education, Completed primary school, Completed secondary school, Completed post-secondary education
10.	Rating of male partner’s support	Little support, Adequate support
11.	Residence	Rural, Urban
12.	History of intimate partner violence	Yes, No
13.	History of stressful life events	Yes, No

S/N	Parameter	Response
1.	Status	First trimester, Second trimester, Third trimester, 6 weeks or less after delivery, More than 6 weeks after delivery
2.	Number of children	…………………………………….
3.	Number of abortions in the past	…………………………………….
4.	Experienced complications during pregnancy	Yes, No
5.	If yes, mention the complications	Malaria, Gestational diabetes, Pre-eclampsia, Preterm delivery, Prolonged/obstructed labor, Other (specify) ……………………………………..
6.	Comorbidities during pregnancy (multiple responses)	Diabetes, HIV, Hypertension, Other (specify) …………………….
7.	Experienced complications during postpartum period	Yes, No
8.	Post-delivery complications reported by participants (multiple responses)	Postpartum hemorrhage, Puerperal sepsis, Other (specify) …………………………………
9.	History of mental illness during previous pregnancy	Yes, No
10.	If yes in 9, specify	…………………………………….
11.	Place of delivery	Health Centre III, Health Centre IV, General hospital, Referral, Home delivery, Other (specify) ………………
12.	Mode of delivery	Vaginal delivery, Caesarean delivery, Assisted delivery
13.	Status of mother	Good health state, Fair, Unstable

S/N	Characteristics of the Infant	Responses
1.	Age of infant	………………………………
2.	Sex of infant	Male, Female
3.	Got the preferred sex of infant	Yes, No
4.	Birth weight (kg)	Weight not measured at birth, <2.5, 2.5-3.0, >3
5.	Infant breastfeeds well	All the time, Sometimes, Not at all
6.	Babies health problem reported by mothers	Yes (specify) ……………, No
7.	Health status of infant	Good condition, Mild health problem, On oxygen

S/N	Parameter	Response
1.	I have been able to laugh and see the funny side of things	As much as I always could, Not quite so much now, Definitely not so much now, Not at all
2.	I have looked forward with enjoyment to things	As much as I ever did, Rather less than I used to, Definitely less than I used to, Hardly at all
3.	I have blamed myself unnecessarily when things went wrong	No, never, Not very often, Yes, some of the time, Yes, most of the time
4.	I have been anxious or worried for no good reason	No, not at all, Hardly ever, Yes, sometimes, Yes, very often
5.	I have felt scared or panicky for no very good reason	No, not at all, No, not much, Yes, sometimes, Yes, quite a lot
6.	Things have been getting on top of me	No, I have been coping as well as ever, No, most of the time I have coped quite well, Yes, sometimes I haven’t been coping as well as usual, Yes, most of the time I haven’t been able to cope at all
7.	I have been so unhappy that I have had difficulty sleeping	No, not at all, Not very often, Yes, sometimes, Yes, most of the time
8.	I have felt sad or miserable	No, not at all, Not very often, Yes, quite often, Yes, most of the time
9.	I have been so unhappy that I have been crying	No, never, Only occasionally, Yes, quite often, Yes, most of the time
10.	The thought of harming myself has occurred to me	Never, Hardly ever, Sometimes, Yes, quite often

S/N	Parameter assessed	Not at all (0)	Several days (1)	More than half the days (2)	Nearly every day (3)
1.	Feeling nervous, anxious, or on edge				
2.	Not being able to stop or control worrying				
3.	Worrying too much about different things				
4.	Trouble relaxing				
5.	Being so restless that it is hard to sit still				
6.	Becoming easily annoyed or irritable				
7.	Feeling afraid as if something awful might happen				
	Total score ____	____ +	+ ____	+ ____	+ ____
